# Medicines informal market in Congo, Burundi and Angola: counterfeit and sub-standard antimalarials

**DOI:** 10.1186/1475-2875-6-22

**Published:** 2007-02-22

**Authors:** Maria Cristina Gaudiano, Anna Di Maggio, Emilia Cocchieri, Eleonora Antoniella, Paola Bertocchi, Stefano Alimonti, Luisa Valvo

**Affiliations:** 1 Dipartimento del Farmaco, Istituto Superiore di Sanità, Viale Regina Elena 299, 00161 Roma, Italy

## Abstract

**Background:**

The presence of counterfeits and sub-standards in African medicines market is a dramatic problem that causes many deaths each year. The increase of the phenomenon of pharmaceutical counterfeiting is due to the rise of the illegal market and to the impossibility to purchase branded high cost medicines.

**Methods:**

In this paper the results of a quality control on antimalarial tablet samples purchased in the informal market in Congo, Burundi and Angola are reported. The quality control consisted in the assay of active substance by means of validated liquid chromatographic methods, uniformity of mass determination, disintegration and dissolution tests. Moreover, a general evaluation on label and packaging characteristics was performed.

**Results:**

The results obtained on thirty antimalarial tablet samples containing chloroquine, quinine, mefloquine, sulphadoxine and pyrimethamine showed the presence of different kinds of problems: a general problem concerning the packaging (loose tablets, packaging without Producer name, Producer Country and sometimes without expiry date); low content of active substance (in one sample); different, non-declared, active substance (in one sample); sub-standard technological properties and very low dissolution profiles (in about 50% of samples). This last property could affect the bioavailability and bioequivalence in comparison with branded products and could be related to the use of different excipients in formulation or bad storage conditions.

**Conclusion:**

This paper evidences that the most common quality problem in the analysed samples appears to be the low dissolution profile. Here it is remarked that the presence of the right active substance in the right quantity is not a sufficient condition for a good quality drug. Dissolution test is not less important in a quality control and often evidences *in vitro *possible differences in therapeutic efficacy among drugs with the same active content. Dissolution profile can be dramatically affected by the choice of excipients in the oral solid formulation and, in many cases, is out of specifications due to the absence of formulation studies by producers of developing countries.

## Background

One of the effects of poverty is that the medicines market in developing countries consists essentially of illegal vendors. The economic restrictions, the weak drug-regulatory systems and the insufficient controls on production, distribution and importation promote a rise of the illegal medicines market [1]. Moreover, the necessity of lowering the treatment costs induces the diffusion in the market of loose medicines, without the original primary packaging, of which, in many cases, the producer name and country, the batch number and the expiry date are not available, thus preventing any possible control of the origin and the quality of the drug, as recommended by the World Health Organization (WHO) [2]. Furthermore, storage and selling conditions are inadequate: in many developing countries, drugs are maintained at high temperature and humidity, not in the original packaging and not protected from the sun. These conditions accelerate the drug degradation process with, in some cases, a lowering of the active substance strength and an increase of degradation products and, possibly, of toxicity [3]. Moreover, storage conditions in tropical regions could affect the drug release profile, as evidenced in medicines from the Tanzanian market [4]. The low quality (sub-standard) medicines and the presence of counterfeits, together with a poor adherence to therapy by the patient, are important causes of death in developing countries [5,6]. In clinical trials, the therapies are well-monitored and the medicines are of good guaranteed quality, but the real therapeutic conditions are very different. Most individuals buy cheap medicines in the informal/illegal market without taking into consideration the risk of it. On the other hand, in most of sub-Saharan countries, the patients have no choice, because the only chance to purchase medicines, when they exist, is through the informal market. This situation favours the development of the illegal traffic of counterfeits. The evaluation of counterfeit and sub-standard medicines in developing countries is about 25% of marketed medicines but, in some countries, this percentage may increase up to 60% [7]. WHO defines a counterfeit medicine as one which is deliberately and fraudulently mislabelled with respect to identity and/or source. Counterfeiting can apply to both branded and generic products and counterfeit products may include products either with correct ingredients, with the wrong ingredients, without any active ingredients, with incorrect quantity of active ingredients or with fake packaging [8]. In developing countries, most counterfeit are of life-saving medicines such as antibiotics, antimalarials, anti-tuberculosis and antiretroviral drugs [9-17]. Every year many cases of counterfeit essential drugs causing therapy inefficacy, development of drug resistance and sometimes death are reported [18-21]. Considering the WHO definition of a counterfeit medicine, how should one consider medicines in plastic bags or enveloped in pieces of paper without any producer name, batch number or expiry date, being sold by non-authorized vendors? What is the real quality of these medicines? What are the real strength, the impurities quantity, the pharmaceutical-technological properties and the bioavailability of these medicines?

In this paper the results of a quality control performed on antimalarial tablets purchased by random sampling in the illegal/informal market in the capitals of Congo, Burundi and Angola are reported. The assay of the active substances was performed by reversed phase liquid chromatography (RP-LC). The uniformity of mass determination, the tablet disintegration and dissolution tests were also carried out. The last was performed to obtain *in vitro *information on possible bioavailability problems [22].

## Methods

### Medicinal samples

Commercial tablet samples of quinine, sulphadoxine and pyrimethamine, chloroquine, mefloquine were purchased from illegal vendors or in small informal pharmacies in Goma (Congo), Bujumbura (Burundi) and Luanda (Angola). Different samplings were performed in different places of these cities with the aid of local health professionals. For each sample the site of purchase, the cost, the environmental conditions (e.g. open stall, without protection from sun, etc.), the product name, the strength, the producer name and country (if available), the batch number and the expiry date (if available), was recorded. The samples were then sent by air to the Istituto Superiore di Sanità in Italy, where analytical controls were performed on thirty antimalarial tablet samples. Italian commercial antimalarial tablet samples were used for comparison in dissolution tests and in the analytical method development and validation. Quinine 250 mg tablets (Nova Argentia, Milano, Italy), chloroquine 250 mg tablets (Bayer AG Leverkusen, Germany), mefloquine 250 mg tablets (Roche S.p.A. Milano, Italy) were purchased from the Italian national market. Sulphadoxine and pyrimethamine (500 mg/25 mg) tablets (Roche Pharma, Reinach, Suisse) were purchased from the Vatican State Pharmacy.

### Chemicals

Chloroquine diphosphate salt (purity>98%), quinine sulphate salt USP (purity = 99.8%) and pyrimethamine (purity>99%) used in standard preparations were purchased from Sigma Chemical Company (St. Louis, MO, USA). Mefloquine hydrochloride and sulphadoxine reference standards were obtained from the European Pharmacopoeia (EDQM, Strasbourg, France). Potassium dihydrogen phosphate was from ICN Biomedicals Inc. (Ohio, USA), 85% phosphoric acid was from Friedel-de Haen GmbH (Germany); 1-pentane-sulfonic acid sodium salt and 99.5% triethylamine were from Sigma-Aldrich GmbH (Steinthem, Germany), HPLC-grade methanol and acetonitrile were from Baker (Deventer, Holland). All other reagents were of analytical grade.

### Chromatographic analysis

The chromatographic equipment consisted of a Series 1100 HPLC system with an automatic injector and a photo-diode array detector (Agilent Technologies Deutschland GmbH, Waldbronn, Germany). For data collection and calculation Chemstation software was used (Agilent Technologies). For the analysis of quinine, chloroquine and mefloquine tablets, a single method, previously validated, was employed [23]. Samples were prepared by suspending the suitable quantity of powder from tablets in phosphate buffer (for chloroquine) and in methanol (for quinine and mefloquine) to obtain, after centrifugation, a final concentration of 0.1 mg/ml in active substance. Quantification of medicinal samples was obtained in triplicate analysis by comparison with standard solutions prepared in triplicate. The chromatographic column was a Symmetry C18, 75 mm × 4.6 mm i.d., 3.5 μm particle size (Waters Corporation, Massachussetts, USA) thermostated at 30°C. The detection wavelength was 230 nm and the injection volume was 10 μl. Mobile phase A was a buffer consisting of 50 mM potassium dihydrogen phosphate, 7 mM 1-pentane-sulfonic acid sodium salt and 0.1% v/v triethylamine. The pH was adjusted to 2.9 ± 0.1 with phosphoric acid before bringing to volume. Mobile phase B was acetonitrile. The elution was a gradient delivered at 1 ml/min as follows: 0–5 min: from 90% A to 70% A, 5–8 min: from 70% A to 50% A, 8–10 min: 50% A. The system was then re-equilibrated to 90% A.

For the analysis of sulphadoxine and pyrimetamine fixed dose composition tablets, the method reported in the United States Pharmacopeia, slightly modified, was employed [24]. The method was re-validated in terms of linearity, precision and accuracy, as reported in Table 1. Samples were prepared by sonicating a suitable quantity of powder from tablets in 35 ml of acetonitrile and by adding 65 ml of 1% v/v acetic acid solution. After filtration samples were opportunely diluted with the same solvent mixture to obtain a final concentration of 0.05 mg/ml and 0.125 mg/ml in sulphadoxine and pyrimethamine, respectively. Quantification of medicinal samples was obtained in triplicate by comparison with standard solutions prepared in triplicate at the test concentration. The chromatographic column for sulphadoxine and pyrimethamine analyses was a C18 Supelcosil ABZ+Plus, 250 mm × 4.6 mm i.d., 5 μm particle size (Supelco, Bellefonte, USA) thermostated at 25°C. The detection wavelength was 254 nm and the injection volume was 10 μl. Mobile phase was a mixture of 1% v/v acetic acid: acetonitrile (64:36 v/v) delivered at 1 ml/min.

**Table 1:** Validation data of the LC method for sulphadoxine and pyrimethamine tablets analysis.

**Linearity**	**Sulphadoxine**	**Pyrimethamine**
Range	10–200%	10–200%
Equation	Y = 26873 X - 3	Y = 19603 X + 2
r^2^	0.99998	0.99994
**Intra-day Precision**		
MEAN_n = 6 _(%RSD)	97.2% (0.6)	101.1% (0.5)
MEAN_n = 6 _(%RSD)	96.8% (0.4)	102.9% (0.4)
MEAN_n = 6 _(%RSD)	98.0% (1.5)	103.9% (0.7)
**Inter-day Precision**		
MEAN_n = 3 _(%RSD)	97.3% (0.6)	102.6% (1.4)
**Accuracy**		
Linear equation (measured % *vs *added %)	Y = 1.05 X + 97.9	Y = 1.01 X + 100.7
r^2^	0.998	0.99994

### Other quality control tests

The Uniformity of Mass was determined on each sample according to the European Pharmacopoeia [25]. Disintegration test was performed following the method and limits reported in the European Pharmacopoeia [26] by using a PTZ AUTO disintegration apparatus (Pharma Test Apparatebau GmbH, Hainburg, Germany). Six tablets of each sample were placed inside the disintegration apparatus filled with distilled water at 37°C and the disintegration was verified after 15 minutes. For sugar coated tablets disintegration was verified after 60 minutes as indicated in the European Pharmacopoeia. Dissolution Test was performed following the specific methods described in the United States Pharmacopoeia [24] by using an AT7 Smart dissolution apparatus (Sotax Italia, Milano, Italy) connected with a Lambda 26 spectrophotometer (PerkinElmer, Wellesley, USA). For quinine tablets a dissolution medium consisting of 0.1N hydrochloric acid was employed, for chloroquine the medium was distilled water and for sulphadoxine and pyrimethamine tablets phosphate buffer at pH = 6.8 was used. The dissolution profiles for chloroquine and quinine were obtained by measuring the absorbance at 343 nm and 248 nm, respectively. For sulphadoxine and pyrimethamine the dissolution profiles were obtained by LC assay.

## Results and Discussion

Information about the vendor, the price and the storage conditions were obtained from the filled in form attached to each sample. In more than 50% of cases, the medicines were sold loose, without the original primary packaging, into little plastic bags with the name of the active ingredient, the strength, the expiry date and, only in some cases, the producer name and country written by pen. In 25% of the analysed samples the expiry date was not available. Moreover, the storage conditions reported in the form were inadequate (open stall, without protection from sun, hand bags).

**Figure 1:**
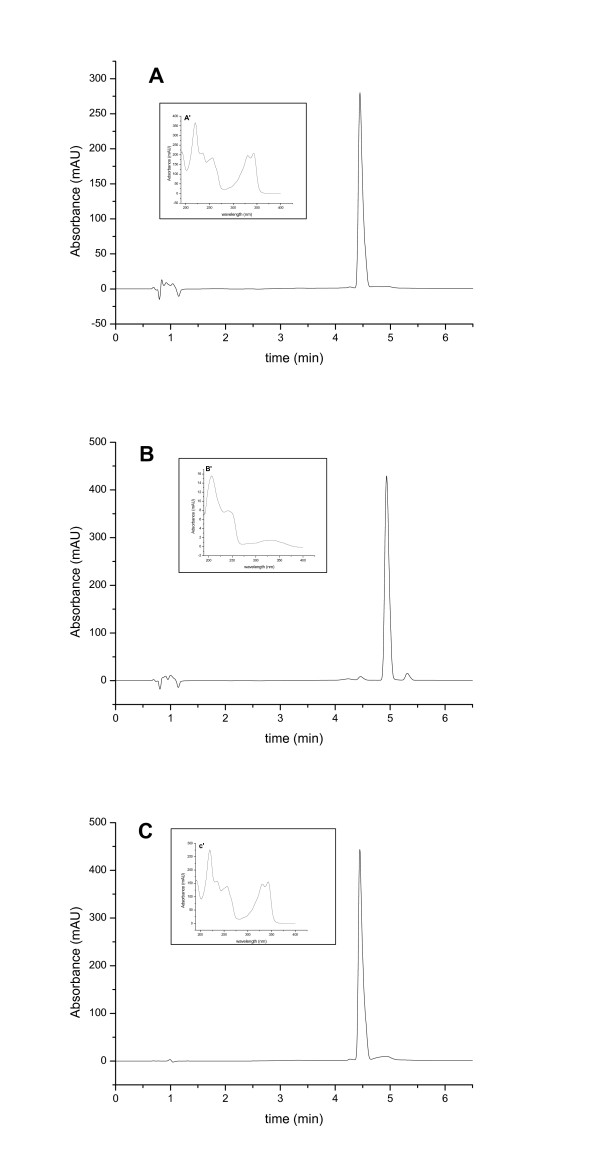
Chromatographic profiles of tablets sample Qb5, purchased as quinine, 300 mg, from Burundi (A), of quinine sulphate standard (B) and of chloroquine diphosphate standard (C). The retention time and the UV-absorption spectrum recorded in the peak apex (reported in the inset), clearly indicates that the sample is chloroquine.

The cost of twenty tablets was about 1–4 USD (United States Dollars) for quinine, 0.2–2 USD for chloroquine, 9–15 USD for mefloquine and 1.5–3 USD for sulphadoxine and pyrimethamine fixed dose composition. In general, from data reported in each form, it was observed that the price of medicines in small pharmacies is not higher than that in the illegal market; on the contrary, sometimes the illegal market sells the same drug at higher price. On the other hand, it should be considered that the so called "small pharmacies" are not, in many cases, legally recognized. Moreover, in both cases no difference in the probability to buy a sample constituted by either loose tablets or tablets packaged in the original blister was found. Concerning the countries of origin, Indian and local products are the more represented in the informal market. Interesting is the case of sample named Qc1, Quinine from the Congo market: this medicine was illegally sold, being a not saleable sample of the "Essential Drugs Programme-WHO", coming from an International Cooperation programme. This event is not infrequent: in many developing countries a percentage of food and drugs donated by International Cooperation reaches the illegal market. An other interesting specimen was the sample named SPc19, sulphadoxine and pyrimethamine tablets produced in China and purchased from Congo: the absence of the expiry date on blister and other characteristics, as the producer name that was similar, but not identical, to that of a real Chinese producer, induced to consider this sample as a counterfeit. The samples were analysed to evaluate their quality. The assay of active substance was performed by validated HPLC methods. Moreover, the uniformity of mass and the disintegration performances were evaluated. Finally, the dissolution profiles were evaluated to obtain some information about possible bioavailability and bioequivalence problems with respect to some commercial European products. In Table 2 the results obtained for each sample are reported. The samples were considered "in specifications" (IS) when the assay for the active substance was in the 90–110% range, based on the larger specifications among those reported in the US, British, European and International Pharmacopeias [24,27-29]. The assay on the active substance evidenced that one sample of quinine (Qc17) was out of specifications (OOS) with a lower amount of active substance (88.6%) and in an other case the active substance found was different from that declared (sample Qb5). The chromatographic retention time (R.T.) of the peak and the UV absorption spectrum allowed to establish that the active was chloroquine instead of quinine. The use of the same analytical method to analyse both kind of samples allowed an easy identification of the wrong ingredient. Moreover, the quantity of active compound was also found different from the one declared (82.4%). This is a typical example of counterfeit medicine characterized by wrong active and wrong quantity. Moreover, considering the different antimalarial efficacy of these two actives and considering that in Burundi, where this counterfeit medicine was purchased, the more common form of malaria is caused by chloroquine-resistant *Plasmodium falciparum*, dispensing this medicine to a patient can give rise to inefficacy and even to death. Figure 1 reports the chromatographic profiles and the absorption spectra of quinine sample Qb5 purchased in the informal market in Burundi (A), standard quinine sulphate (B) and standard chloroquine diphosphate (C). The different R.T. (4.5 instead of 4.9 minutes), the UV spectra and the absence of the two characteristic impurities at R.T. 4.45 and 5.35 minutes, clearly indicate that the sample was not quinine. After the identification of the real active substance, the extraction was repeated to evaluate the quantity of chloroquine in the tablet. The strength was about 250 mg, characteristic of chloroquine tablets, instead of 300 mg, as declared. The substitution of quinine with the cheaper chloroquine appears to be an usual practice in African countries as reported also by other authors [21]. Concerning the uniformity of mass, two samples were found OOS. Disintegration performances for all samples were found IS, as reported in Table 2.

**Table 2:** Analytical results of the antimalarial samples purchased from the informal market in Congo, Burundi and Angola.

**Sample**^1^	**Packaging/Exp. Date**^2^	**Origin**^3^	**% Assay (%RSD)**	**Uniformity of Mass**	**Disintegration Test**	**% Dissolved^4 ^(%RSD)**
Qc1	In the "Essential Drugs Programme-WHO" pot/02-2007	Cyprus	97.0 (0.9)	IS	IS	98 (1)
Qc4	loose tablets/08-2008	Congo	99.8 (0.4)	IS	IS	96 (1)
Qc6	loose tablets/07-2007	Holland	102.7 (0.7)	IS	IS	100 (2)
Qc10	loose tablets/04-2008	Congo	100.3 (0.8)	IS	IS	96 (2)
Qc11	loose tablets/10-2007	Congo	99.5 (0.7)	**OOS**	IS	96 (1)
Qc12	loose tablets/08-2006	Congo	98.5 (0.2)	IS	IS	101 (1)
Qc14	loose tablets/NA	NA^4^	96 (2)	IS	IS	**75 **(5) **OOS**
Qc17	loose tablets/NA	NA	**88.6 **(0.6) **OOS**	IS	IS	**58 **(7) **OOS**
Qc18	loose tablets/NA	NA	97.4 (0.8)	IS	IS	**72.3 **(0.6) **OOS**
Qc20	in blister/01-2007	India	95.2 (0.4)	IS	IS	99 (4)
Qb1	loose tablets/11-2009	NA	99.0 (0.5)	IS	IS	96 (1)
Qb4	in blister/05-2008	Burundi	99 (1)	IS	IS	101 (2)
Qb5	loose tablets/05-2008	NA	**Different Active**	**OOS**	IS	**30 **(8) **OOS**
Qb6	loose tablets/03-2008	NA	95.9 (0.3)	IS	IS	88 (4)
Qa3	in blister and secondary packaging/08-2008	India	97.4 (0.8)	IS	IS	**50 **(5) **OOS**
Qa5	in blister/10-2007	India	98 (1)	IS	IS	98 (2)
Ma4	in blister/06-2006	Brazil	102.6 (0.9)	IS	IS	Not evaluated
Ma6	in blister and secondary packaging/08-2007	Cyprus	102.4 (0.8)	Not evaluated	Not evaluated	Not evaluated
Ca1	in blister and secondary packaging/08-2007	India	102 (2)	IS	IS	**26 **(3) **OOS**
Ca2	in blister/08-2007	India	102.8 (0.7)	IS	IS	**75 **(7) **OOS**
						**% Dissolved^5 ^(%RSD)**
SPc2	tablets loose/11-2006	Malta	S = 94.8 (0.6)	IS	IS	S = 79.5 (0.2)
			P = 99.1 (0.1)			P = 73 (2)
SPc3	in blister and secondary packaging/06-2007	India	S = 96.3 (0.3)	IS	IS	S = 97 (5)
			P = 100.3 (0.2)			P = 67.9 (0.6)
SPc5	in blister and secondary packaging/02-2007	India	S = 98 (1)	IS	IS	S = 99 (1)
			P = 100.9 (0.6)			P = 72 (1)
SPc7	tablets loose/10-2008	Cyprus	S = 95.9 (0.6)	IS	IS	S = 95 (3)
			P = 97.9 (0.4)			P = 73 (3)
SPc8	tablets loose/NA	NA	S = 94.0 (0.2)	IS	IS	**S = 57 **(3) **OOS**
			P = 94.1 (0.6)			**P = 24 **(1) **OOS**
SPc9	in blister and secondary packaging/06-2007	India	S = 96 (1)	IS	IS	S = 84 (3)
			P = 102.7 (0.1)			**P = 52 **(2) **OOS**
SPc13	loose tablets/NA	NA	S = 93.3 (0.4)	IS	IS	S = 80.6 (0.7)
			P = 104.6 (0.6)			**P = 53 **(7) **OOS**
SPc15	loose tablets/NA	NA	S = 95 (1)	IS	IS	S = 72 (1)
			P = 100.7 (0.9)			**P = 45 **(3) **OOS**
SPc16	loose tablets/NA	NA	S = 97.3 (0.2)	IS	IS	S = 95 (1)
			P = 100.8 (0.5)			**P = 55 **(5)**OOS**
SPc19	in blister/NA	China	S = 93.8 (0.4)	IS	IS	**S = 47 **(2)**OOS**
			P = 99.1 (0.8)			**P = 18.6 **(0.6)**OOS**

^1^**Qc#, Qb#, Qa#**: Quinine tablet samples from Congo, Burundi and Angola, respectively

**Ca#**: Chloroquine tablet samples from Angola

**Ma#**: Mefloquine tablet samples from Angola

**SPc#**: Sulphadoxine and pyrimethamine tablet samples from Congo

^2 ^The samples were purchased in August 2005

^3 ^Declared Producer Origin Country

^4 ^USP tolerances for quinine sulphate and chloroquine phosphate tablets: not less than 75%(Q) (equivalent to "not less than 80%") of the labelled amount is dissolved in 45 minutes.

^5 ^USP tolerances for sulphadoxine and pyrimethamine tablets: not less than 60%(Q) (equivalent to "not less than 65%") of the labelled amount is dissolved in 30 minutes.

**NA**: not available

**IS**: in specifications

**OOS**: out of specifications

**Figure 2:**
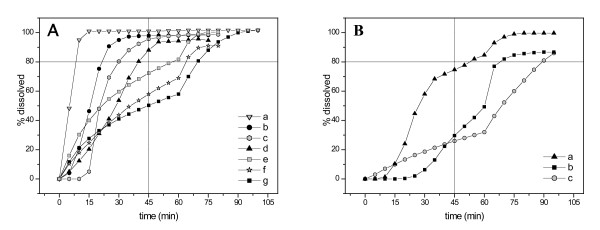
Dissolution profiles of representative samples of quinine (A) and chloroquine (B) tablets. 2A: samples Qc12 (a), Qa5 (b), Qc11 (c), Qb6 (d), Qc18 (e), Qc17 (f), Qa3 (g); 2B: samples Ca2 (a), Qb5 (b), Ca1 (c).

**Figure 3:**
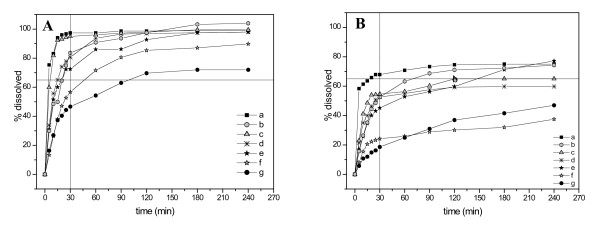
Dissolution profiles of representative samples of sulphadoxine (A) and pyrimethamine (B) fixed dose composition tablets. SPc3 (a); SPc9 (b); SPc16 (c), SPc13 (d), SPc15 (e), SPc8 (f), SPc19 (g).

Dissolution was performed to obtain information about the possible differences in the bioavailability of the antimalarial samples. For quinine sulphate and chloroquine phosphate tablets dissolution specification was above 75%(Q) (equivalent to "not less than 80%") of the labelled amount should be dissolved in 45 minutes; for sulphadoxine and pyrimethamine tablets dissolution specification was above 60%(Q) (equivalent to "not less than 65%") of the labelled amount should be dissolved in 30 minutes, as reported in the US Pharmacopeia specific monographs [24]. The results of the dissolution tests are reported in Table 2 and representative dissolution profiles are shown in Figure 2 and Figure 3. For some quinine tablets an immediate complete dissolution was observed (curve a, Figure 2A) while, for other samples, sigmoidal and biphasic behaviour was found (curves c, e, f and g, Figure 2A). Four samples were found OOS, as reported in Table 2. For chloroquine tablets, three very different biphasic behaviours, all OOS, were observed. It should be recalled that the sample Qb5, reported here in the chloroquine dissolution profiles (curve b, Figure 2B), had been sold as quinine. Moreover, sample Ca1, that was IS for the assay, showed a very poor dissolution profile (curve c, Figure 2B). For sulphadoxine two samples were OOS but, in general, more regular trends were observed, as indicated in Figure 3A (curves g and f). The same samples analysed for dissolution profile of pyrimethamine evidenced very low percentages of active substance dissolved: six out of ten samples were found OOS (curves b-g, Figure 3B) and two of them with a very low release percentage (24% and 19% dissolved after 30 minutes). Pyrimethamine is practically insoluble in water [26] and this characteristic dramatically affects the dissolution profile, as often observed for slightly soluble or insoluble active substances. These results led to the hypothesis that a problem in the bioavailability may be due to the formulations employed and/or to the storage conditions [4]. In fact, in local products or in products obtained from other developing Countries, the excipients in the formulation of branded products are often substituted by cheaper ones without previously performing bioavailability or bioequivalence studies or, at least, comparative *in vitro *studies to verify the influence of the different formulation on the dissolution profile. Moreover, the possible existence of polymorphic forms of the active could also affect the bioavailability of the formulation as well as its dissolution profile.

## Conclusion

The results obtained from the analysed samples show three different kinds of problems: (i) the presence of a low quantity of active substance, observed in one sample; (ii) the substitution of an active substance by a different one, observed in one sample, and (iii) the OOS results concerning the dissolution profile, a parameter that correlates with bioavailability. This kind of OOS was observed in 13 samples out of 28 (46% of cases). Moreover, the high RSD% values observed in some dissolution tests indicate a large variability in the production process, that is non-controlled and non-"Good Manufacturing Practices". The dissolution test is a technological test not usually performed in the quality control of samples from developing countries market. The results reported here indicate that in many cases even samples containing the right quantity of active substance could do not show the same therapeutic properties in terms of bioavailability. In fact, assuming a medicine with a very high or very low absorption rate in the body in comparison with the branded medicine for which *in vivo *efficacy was demonstrated, could affect the good result of therapy. The change in excipients could dramatically affect the *in vivo *efficacy if a bioequivalence study or, at least, an *in vitro *study such as the dissolution test was not performed. Moreover, the OOS results were obtained for pyrimethamine probably because of its poor solubility in water. This argument will be investigated for other life-saving medicines containing low water-soluble active substances marketed in developing countries. Generally, counterfeits and sub-standards are considered an economic and health problem mainly owing to lower strength, absence of active substance or presence of a different active. The results here reported evidenced as the legal or illegal production in developing countries could also involve problems of bioavailability and bioequivalence of medicinal products. When bioequivalence studies are not performed, the assumption that a drug with the right content of active substance is "in standard" is not always correct, especially with low soluble actives. In these cases, the health professional should consider that the therapeutic response could be very different from that expected.

## Authors' contributions

MCG: conception and design of the study, analysis and interpretation of data, drafting of manuscript.

ADM and EC: sample preparation, data collection and analysis of data.

EA and PB: dissolution test measurements supervision and data interpretation.

SA: sample preparation and data collection.

LV: supervision on the progress of the study and revision of the manuscript.

All authors read and approved the final manuscript.
